# Establishment and evaluation of a training course in advanced laparoscopic surgery based on human body donors embalmed by ethanol-glycerol-lysoformin fixation

**DOI:** 10.1007/s00464-020-07523-6

**Published:** 2020-05-22

**Authors:** Johannes Ackermann, Thilo Wedel, Heiko Hagedorn, Nicolai Maass, Liselotte Mettler, Tillmann Heinze, Ibrahim Alkatout

**Affiliations:** 1 Department of Gynecology and Obstetrics, Kiel School of Gynaecological Endoscopy, University Hospitals Schleswig-Holstein, Campus Kiel, 24105 Kiel, Germany; 2 Institute of Anatomy, Center of Clinical Anatomy, Christian-Albrechts-University Kiel, Kiel, Germany

**Keywords:** Ethanol-glycerol-lysoformin fixation, Body donors, Laparoscopy, Surgical education, Clinical anatomy

## Abstract

**Background:**

Education of clinical anatomy and training of surgical skills are essential prerequisites for any surgical intervention in patients. Here, we evaluated a structured training program for advanced gynecologic laparoscopy based on human body donors and its impact on clinical practice.

**Methods:**

The three-step training course included: (1) anatomical and surgical lectures, (2) demonstration and hands-on study of pre-dissected anatomical specimens, and (3) surgical training of a broad spectrum of gynecological laparoscopic procedures on human body donors embalmed by ethanol-glycerin-lysoformin. Two standardized questionnaires (after the course and 6 months later) evaluated the effectiveness of each of the training modules and the benefits to surgical practice.

**Results:**

Eighty participants took part in 6 training courses using a total number of 24 body donors (3 trainees/body donor). Based on a 91.3% (73/80) response rate, participants rated high or very high the tissue and organ properties of the body donors (*n* = 72, 98.6%), the technical feasibility to perform laparoscopic surgery (*n* = 70, 95.9%), and the overall learning success (*n* = 72, 98.6%). Based on a 67.5% (54/80) response rate at 6 months, participants rated the benefit of the course to their daily routine as very high (mean 80.94 ± 24.61%, *n* = 53), and this correlated strongly with the use of body donors (*r* = 0.74) and the ability to train laparoscopic dissections (*r* = 0.77).

**Conclusions:**

This study demonstrates the technical feasibility and didactic effectiveness of laparoscopic training courses in a professional and true-to-life setting by using ethanol-glycerol-lysoformin embalmed body donors. This cost-efficient fixation method offers the option to integrate advanced surgical training courses into structured postgraduate educational curricula to meet both the technical demands of minimal invasive surgery and the ethical concerns regarding patients´ safety.

The training of surgical skills in training models is a fundamental part of surgical education in laparoscopic surgery since its first beginning [1]. In respect to the increasing limitations of human and financial resources as well as higher ethical standards in modern medicine, new training concepts are essential in surgical education [2]. The knowledge of surgically relevant topographic anatomy is becoming more important with the increasing complexity of minimally invasive procedures and the growing expectations for surgeons that cannot be easily met due to long learning curves [3]. These growing demands on specialist surgeons requested by the respective professional societies is countered by the lack of firm integration of clinical anatomy into both undergraduate curricula and postgraduate medical education, and the lack of valid training concepts [4–6]. In the interest of patients´ safety, traditional learning concepts such as learning by watching and learning by doing ("see one, do one, teach one") [7] can no longer be regarded as the answer to this issue [5].

Traditionally, laparoscopic training is performed by using pelvitrainers, virtual trainers, cadavers of animals, as well as living animals [8, 9]. Each of these training methods has its own focus and justification in the training of laparoscopic surgery [10], but also confined by natural limitations, such as lack of resemblance of human anatomy, missing tissue feedback or unreal tissue properties [11, 12]. In particular, the practical learning of clinical anatomy, although considered to be essential for surgeons, is usually missing within the scope of classical laparoscopic training tools [12].

In this context, a promising alternative is the surgical training on human body donors, which overcomes the above-mentioned disadvantages and offers realistic tissue properties and feedback with a true-to-life learning atmosphere, thus representing the gold standard for laparoscopic training [13]. Nearly all laparoscopic procedures can be simulated on body donors in the most realistic available way. Among the different methods available for appropriate fixation of body donors, glycerol-ethanol-lysoformin fixation is a cost-effective and resource-saving option enabling excellent preservation with natural tissue properties [14–16].

The use and suitability of this new fixation technique for laparoscopic procedures in body donors has been described in a previous study [17], which concluded that it permitted laparoscopic surgery in a realistic and practical manner. Based on this promising proof-of-principle study, the use of human body donors embalmed by ethanol-glycerol-lysoformin fixation was introduced in advanced training courses for laparoscopic surgery. The surgical training on body donors was embedded in an integrative training course consisting of anatomical and surgical lectures, and hands-on demonstrations of pre-dissected anatomical specimens of the regions of interest prior to the surgical interventions. The aim of this study was to examine the suitability of body donors for training laparoscopic skills in gynecology and the effectiveness of this tripartite training concept in improving the daily clinical routine of surgeons.

## Material and methods

The study took place at the Kiel School of Gynecological Endoscopy, University Hospitals Schleswig–Holstein, Campus Kiel, Germany, with data collected from 2011 to 2016. Anonymous voluntary course evaluation is standard practice in all courses run by the School and ethical approval was not required after discussion with our Ethics Committee.

### Didactic concept of the tripartite training course

**Fig. 1:**
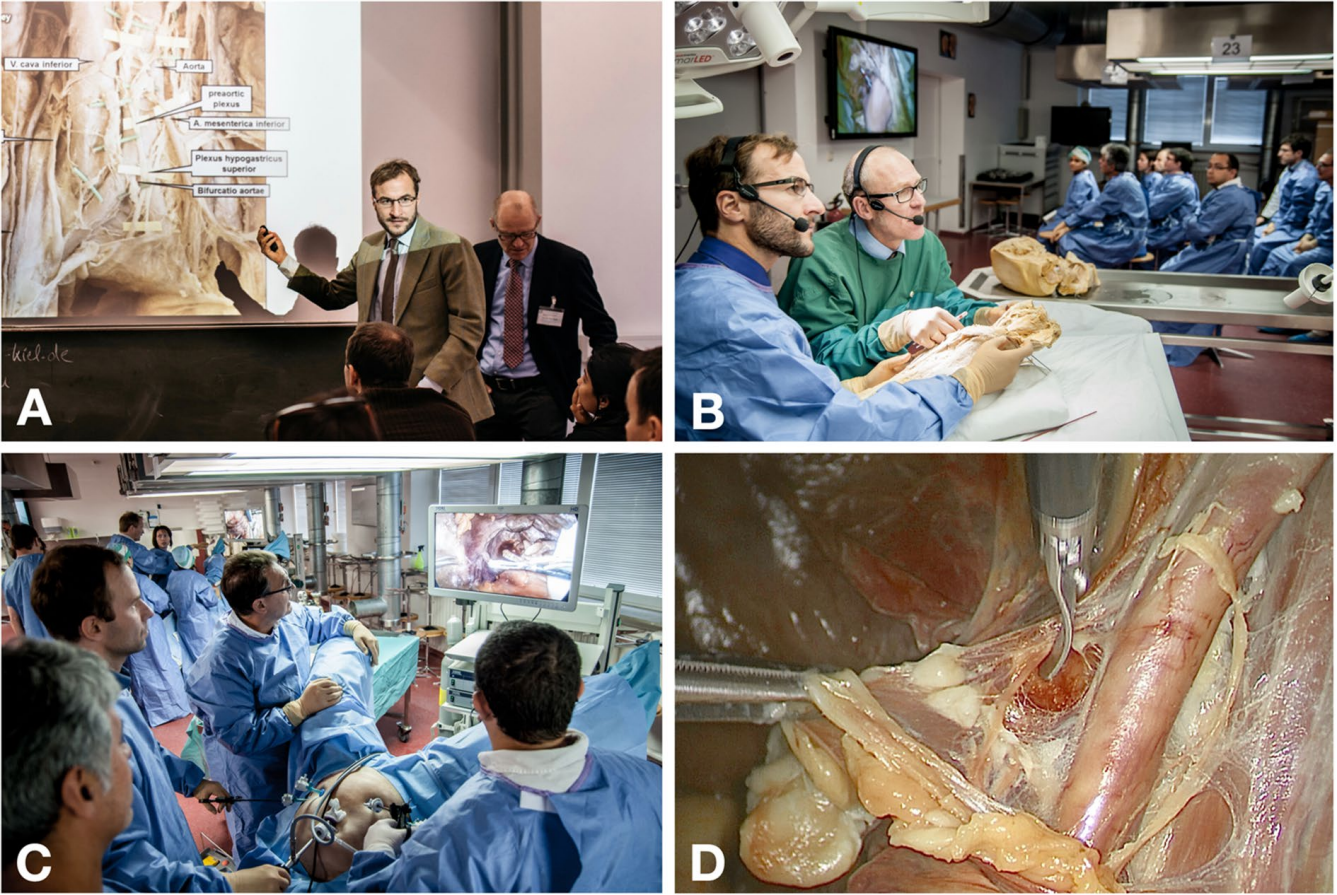
Set-up of the tripartite training course. **A** Anatomical and surgical lectures of the anatomical regions and laparoscopic procedures of interest. **B** Interactive demonstration of pre-dissected anatomical specimens by an anatomist (green surgical gown) and surgeon (blue surgical gown) with subsequent hands-on study by the participants. **C** Laparoscopic surgical training on human body donors. Maximum three trainees with one instructor are working in a surgical set-up equipped with a mobile operating table, laparoscopic tower, instruments and energy devices. **D** Intraoperative situation illustrating pelvic lymph node dissection along the right external iliac vessels (Color figure online)

The advanced laparoscopic surgical training course on human body donors was established in 2011 at the Kiel School of Gynecological Endoscopy, University Hospitals Schleswig–Holstein, Campus Kiel, Germany, together with the Institute of Anatomy, Center of Clinical Anatomy, Christian-Albrechts-University Kiel, Germany and has been certified by the Schleswig–Holstein Medical Association. This postgraduate training course was offered to qualified gynecological laparoscopic surgeons and held on two consecutive days. The three-stage training concept consisted of the following modules (Fig. 1):

#### Anatomical and surgical lectures

A total of 4 h 20 min was allocated for lectures. For each laparoscopic procedure, the corresponding topographic anatomy was illustrated by a clinical anatomist with special emphasis on anatomical landmarks, variations and structures at risk of injury. An experienced surgeon complemented the anatomical presentation by representative videoclips and a stepwise explanation of the intervention and its potential pitfalls (Fig. 1A).

#### Interactive demonstration of pre-dissected anatomical specimens

A total of 1 h 30 min of interactive demonstration of anatomical specimens was allocated during the course. Both the anatomist and surgeon demonstrated pre-dissected formalin-fixed anatomical specimens displaying the relevant topographic anatomy. While the anatomist described the basic anatomical setting (e.g., pelvic organs, fasciae and ligaments, blood and nerve supply, lymphatic drainage), the surgeon illustrated the different approaches, laparoscopic perspectives and procedural steps required for each intervention (Fig. 1B). After the video-transmitted demonstration, the participants were asked to identify and reproduce all anatomical details by hands-on examination of the pre-dissected specimens.

#### Surgical training on human body donors

Participants were allocated 7 h in total for hands-on laparoscopic training on ethanol-glycerol-lysoformin embalmed body donors. Each course was equipped with 4–5 working stations/body donors and a complete operating room set-up including a mobile operating table, laparoscopic tower, instruments and energy devices. In every station, there was a dedicated experienced instructor and three participants. Under supervision, the participants performed surgical procedures of different degrees of complexity in the field of benign gynecology, urogynecology and gynecological oncology. The laparoscopic operations included different types of hysterectomy, adnexectomy, sacrocolpopexy, pectopexy, colposuspension, pelvic and para-aortic lymphadenectomy.

### Pre-dissected anatomical specimens

Anatomical specimens of the regions of interest (female pelvis, retroperitoneum) were obtained from formalin-fixed body donors recruited from the body donation program of the Institute of Anatomy, Christian-Albrechts University Kiel, Germany. Prior to their death, donors consented in writing to the use of their bodies for educational and research purposes. Dissection of specimens focused on those anatomical structures relevant to the laparoscopic procedures. Several specimens were dissected to highlight the different steps and landmarks of each procedure. Special attention was given to structures which are potentially at risk for intraoperative injury, e.g., nerves, blood vessels, ureter, adjacent organs, etc. Care was taken to preserve those structures which are surgically relevant but normally removed in conventional dissected anatomical specimens, e.g., pelvic fasciae, fatty tissue, lymph nodes and vessels, minor blood vessels and small-sized autonomic nerve fibers.

### Body donors for laparoscopic skills training

Body donors were recruited from the same body donation program and embalmed by ethanol-glycerol-lysoformin fixation. Those with advanced stages of arteriosclerosis and previous abdominal surgery were excluded in order to achieve efficient perfusion fixation and optimal conditions for laparoscopic surgery. Explorative laparoscopy was carried out in each body donor prior to the courses to confirm the presence of uterus and adnexa and exclude severe adhesions or other major pathologies.

### Ethanol-glycerol-lysoformin fixation

The detailed fixation procedure has been reported previously [17]. Briefly, body donors were perfused with a fixative solution (70% ethanol, 30% glycerin, 0.3% Lysoformin) administered at a ratio of 0.3 l/kg body weight (15–30 l) via the femoral artery. Perfusion was carried out by alternating cycles of injections (30 min) and breaks (20 min) over a period of about 24 h. Fixed body donors were draped in cloths moistened with a watery solution supplemented with 1% thymol, placed in a sealed plastic bag and stored at 4 °C until use.

### Laparoscopic equipment

Laparoscopic surgery was performed in the dissection hall at the Institute of Anatomy, Christian-Albrechts University Kiel. Body donors were placed on mobile operating tables (Yuno, Maquet Getinge Group, Rastatt, Germany) allowing different positions (e.g., Trendelenburg, anti-Trendelenburg, lateral inclination) in order to properly expose the regions of interest. The laparoscopic equipment (Karl Storz GmbH & Co. KG, Tuttlingen, Germany) included an endoscopy system (2D full HD, IMAGE1-S, 30° optic lens), CO_2_ insufflation (electronic Endoflator 264305 20), a rinsing device (Hamou Endomat 263310 20), standard laparoscopic instruments (Karl Storz Clickline), and image processing devices (Image 1 TC 200, Image 1 H3- Link TC 300). Electrosurgery (BOWA-electronic GmbH & Co. KG, Gomaringen, Germany) was performed with MetraLOOP 520-115, ERGO 310D 775-000 and Bowa Arc 400. 10-mm and 5-mm trocars equipped with inflatable cuffs (Kii Optical, Applied Medical, CA, USA) were used to prevent trocar dislocation. In case the trocar entry sites were not completely gas-tight, they were additionally secured with purse-string sutures (Prolene, Ethicon, Johnson & Johnson Medical).

### Evaluation

**Fig. 2:**
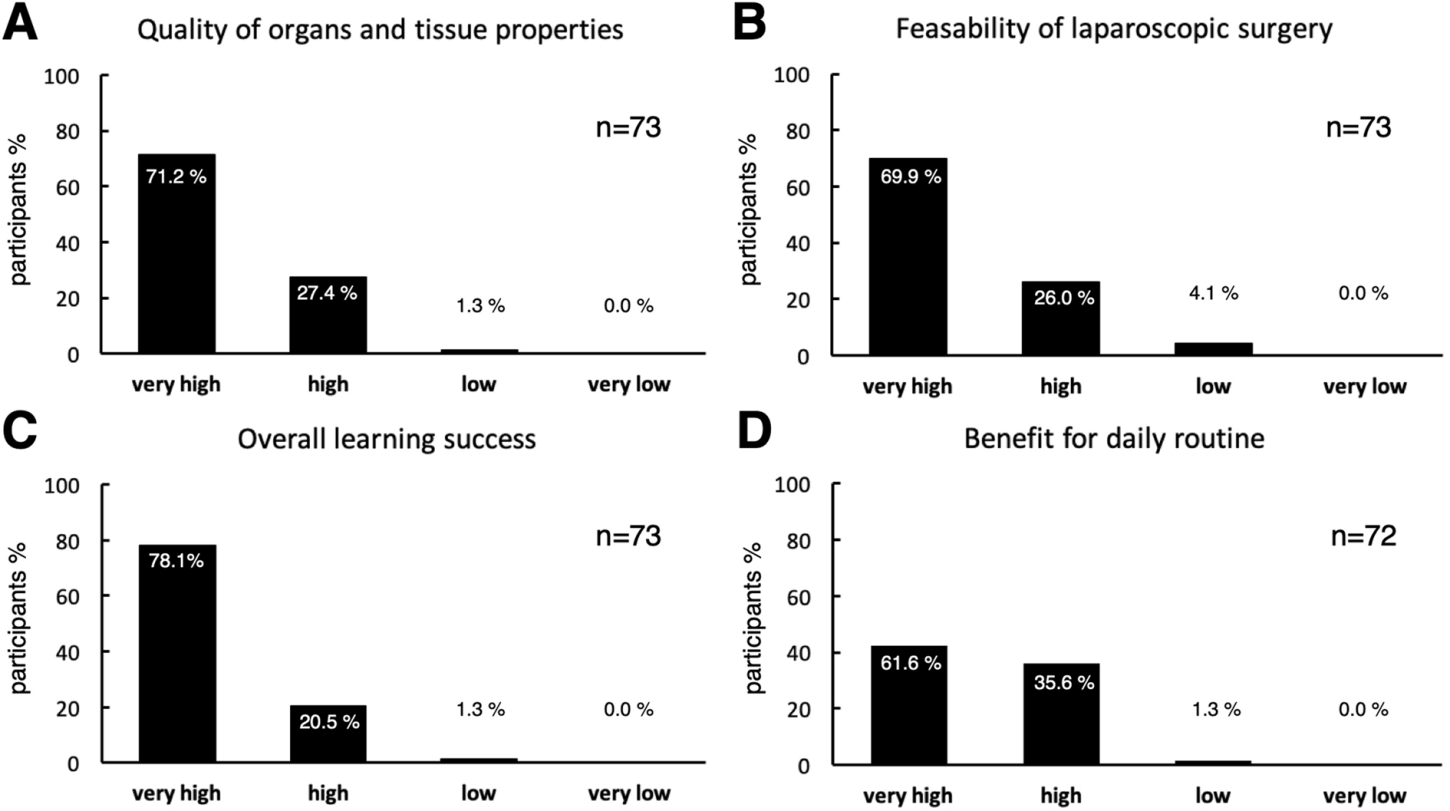
Evaluation of the training course directly after termination (Likert type responses)

A twofold evaluation was carried out by the participants by means of anonymized standardized questionnaires. The first evaluation, which is a standard questionnaire provided by industry partners and sponsors and used in other laparoscopic courses at the institution, was conducted directly after finishing the course and focused primarily on the suitability of body donors for the training of laparoscopic procedures and its impact on practical learning success with Likert scale responses [18] (very high, high, low, very low) (Fig. 2, Table 2). In addition, open questions regarding advantages and disadvantages of the course and suggestions for improvement were given.

**Fig. 3:**
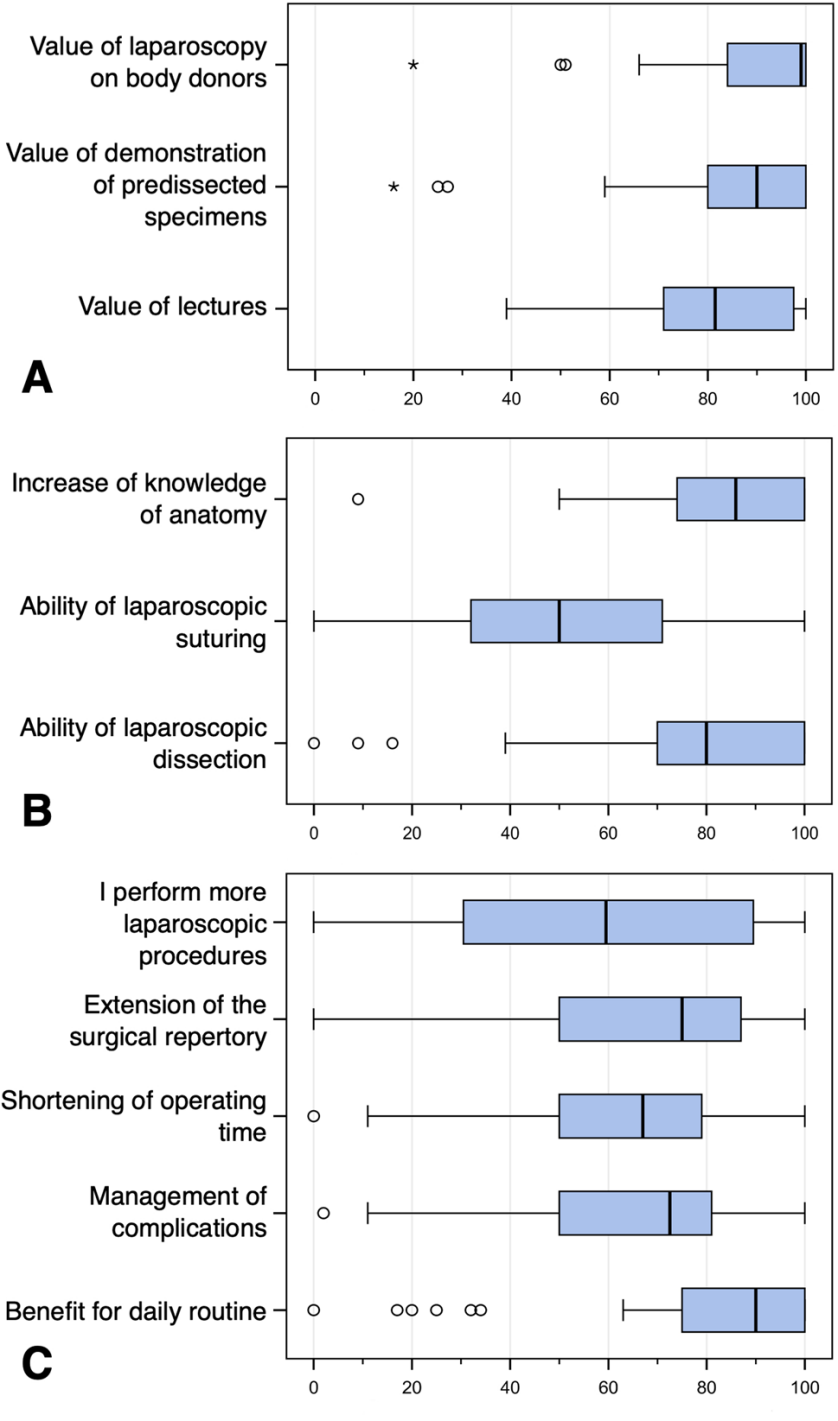
Boxplots of participants’ evaluation of the training course after 6 months. **A** Value of each individual module of the training course. **B** Value of the training course to increase anatomical knowledge and to improve technical laparoscopic skills. **C** Value of the training course to improve laparoscopic procedures (number, repertoire, operating time, management of complications, daily routine)

The second evaluation was performed six months after the course via an online anonymized questionnaire sent by e-mail. It was constructed with the cooperation of a statistician, anatomists and clinicians, and is also used in other laparoscopy courses (without body donors) in a modified form. The questions referred to the didactic value of the different modules of the course and the impact of the laparoscopic skills training on the daily surgical routine (Fig. 3). The answers were recorded by means of a continuous visual analogue scale (VAS) and expressed in percentage (0: not useful; 100: very useful). The questionnaires can be found in the supplementary data.

### Statistical analysis

IBM SPSS Statistics 23 was used for statistical analysis. Quantitative variables were presented descriptively as mean and standard deviation, minimum, maximum, and quartiles, and tested for normality with the Kolmogorov–Smirnov test. For the purposes of describing the VAS values, the following convention was used: < 20, very low; 20 to < 40, low; 40 to < 60, moderate; 60 to < 80, high; 80 to 100, very high.

A correlation analysis was performed to determine the influence of the several parameters of the online evaluation at 6 months post course on the overall benefit of the laparoscopic training course to the daily routine. The Spearman-rho test was used for correlation analysis, when significant deviations from normal distribution were found. Correlation coefficient (*r*) was evaluated as follows: *r* ≤ 0.2, no correlation; 0.2 < *r* ≤ 0.5, weak to moderate correlation; 0.5 < *r* ≤ 0.8, strong correlation; 0.8 < *r* ≤ 1.0, very strong correlation. Matching responses between the two questionnaires was not possible due to anonymization and, therefore, no more correlations were performed. Tests were performed bilaterally and a significance level of 5% was used (*p* < 0.05).

## Results

### Course participants

From 2011 to 2016, 6 courses of advanced laparoscopic surgery in gynecology were conducted, with overall 80 participants. The response rate to the questionnaire at the end of each course was 91.3% (73 out of 80 participants). Fifty-eight participants (79.5%) had over 5 years of professional experience in gynecologic laparoscopic surgery, 13 participants (17.8%) 1–5 years and only one participant (1.4%) less than 1 year. While 48 participants (65.8%) had previously attended one or more laparoscopic training courses, 14 participants (19.2%) participated for the first time and 11 participants (15.1%) gave no information (Table 1).

**Table 1:** Details of course participants (*n* = 73)

	*n* (%)
Years of professional experience	
< 1 year	1 (1.4%)
1–2 years	5 (6.8%)
3–5 years	8 (11.0%)
> 5 years	58 (79.5%)
Not specified	1 (1.4%)
Participation in other laparoscopic training courses	
Yes	48 (65.8%)
No	14 (19.2%)
Not specified	11 (15.1%)

### Evaluation of the body donors and course

The body donors consisted of 24 women, with an age range of 72 to 103 years, and weight 41 to 80 kg. The quality of the organs and the tissue of human body donors embalmed by the ethanol-glycerol-lysoformin fixation was rated as very high by 71.2% of the participants (*n* = 52) and high by 27.4% of the participants (*n* = 20). One participant (1.3%) rated the quality of the organs and of the tissue as low (Fig. 2A).

The feasibility of laparoscopic surgery on these donors was rated by 69.9% of the participants (*n* = 51) as very high and by 26.0% of the participants (*n* = 19) as high. Three participants (4.1%) rated feasibility of laparoscopic surgery on human body donors as low (Fig. 2B).

The overall learning success of the training course on human body donors embalmed by the ethanol-glycerol-lysoformin fixation was rated as very high by 78.1% of the participants (*n* = 57) and high by 20.5% of the participants (*n* = 15). One participant (1.3%) rated the overall learning success of the training course as low (Fig. 2C). The benefit of the training course to the daily routine was rated as very high by 61.6% of the participants (*n* = 45) and as high by 35.6% of the participants (*n* = 26). One participant (1.3%) rated the benefit of the training course on human body donors to the daily routine as low (Fig. 2D).

Furthermore, there was an overwhelming positive opinion about the quality, presentation and specific benefits of the course (Table 2), with 85–97% of participants giving a high or very high evaluation.

**Table 2:** Evaluation immediately post course

Question	Very high	High	Low	Very low	No answer
Quality of theoretical teaching	46	25	0	0	0
Quality of practical teaching	51	19	2	0	1
Benefit of the demonstration of anatomical specimens	55	6	0	0	12
Learning of new knowledge	35	33	3	0	3
Improvement of knowledge	45	26	0	0	2
Presentation of the learning material	53	16	1	0	1
Exchange of experience	55	15	2	0	1

Based on answers to the open questions, the commonest reasons for attending the course was to learn and refresh anatomical knowledge, and desire to attend the highest level of training in laparoscopy. Participants particularly liked the anatomical teaching in theory and practice, the refresher lectures and hands-on practice, as well as the demonstration of the anatomy in pre-dissected specimens. Some commended in the very natural conditions of the body donors, and that theory and practice were linked appropriately.

However, some negative comments included the lack of time and the large differences in the level of experience of the participants. They recommended a similar course focused on beginners, less theory, and only 2 participants per body donor.

### Online evaluation 6 months after course participation

An evaluation of the course was performed six months after each course via an online questionnaire send to course participants via e-mail, to follow up the long-term success of the course for their daily routine. The response rate was 67.5% (54 out of 80 participants). The questions have been rated on a scale bar from 0 (not useful) to 100 (very useful). The results are shown in Fig. 3 (boxplots).

Regarding the relative value of the individual modules on the overall benefit of the course, performing laparoscopy on human body donors was evaluated as particularly useful (mean 89.70 ± 15.89%, *n* = 53). The lectures (mean 80.69 ± 16.19%, *n* = 52) and the demonstration of anatomy on pre-dissected anatomical specimens (mean 85.13 ± 19.35%, *n* = 54) were also evaluated very highly regarding the overall benefit of the entire course (Fig. 3A).

The overall benefit of the laparoscopic training on human body donors to the surgeon’s daily routine was rated as very high (mean 80.94 ± 24.61%, *n* = 53). When asked to rate the benefit of the course in performing more laparoscopic operations, the response was very broad, with a median of 59.50% (mean 57.35 ± 35.17%, *n* = 52). The course was reported as highly useful in extending the surgical repertoire (64.19 ± 32.59%, *n* = 53), the shortening of operation time (mean 62.61 ± 22.73%, *n* = 54) and the management of complications (mean 65.93 ± 24.04%, *n* = 54) (Fig. 3B).

Participants found the course particularly useful for improving their laparoscopic dissection skills (mean 77.94 ± 24.11%, *n* = 54) and their knowledge of anatomy (mean 83.28 ± 17.80%, *n* = 53). The benefit of the training for laparoscopic suturing was rated as moderate (mean 51.63 ± 29.76%, *n* = 54) (Fig. 3C).

### Correlation of different parameters to the overall benefit of the training course to the daily routine

**Fig. 4:**
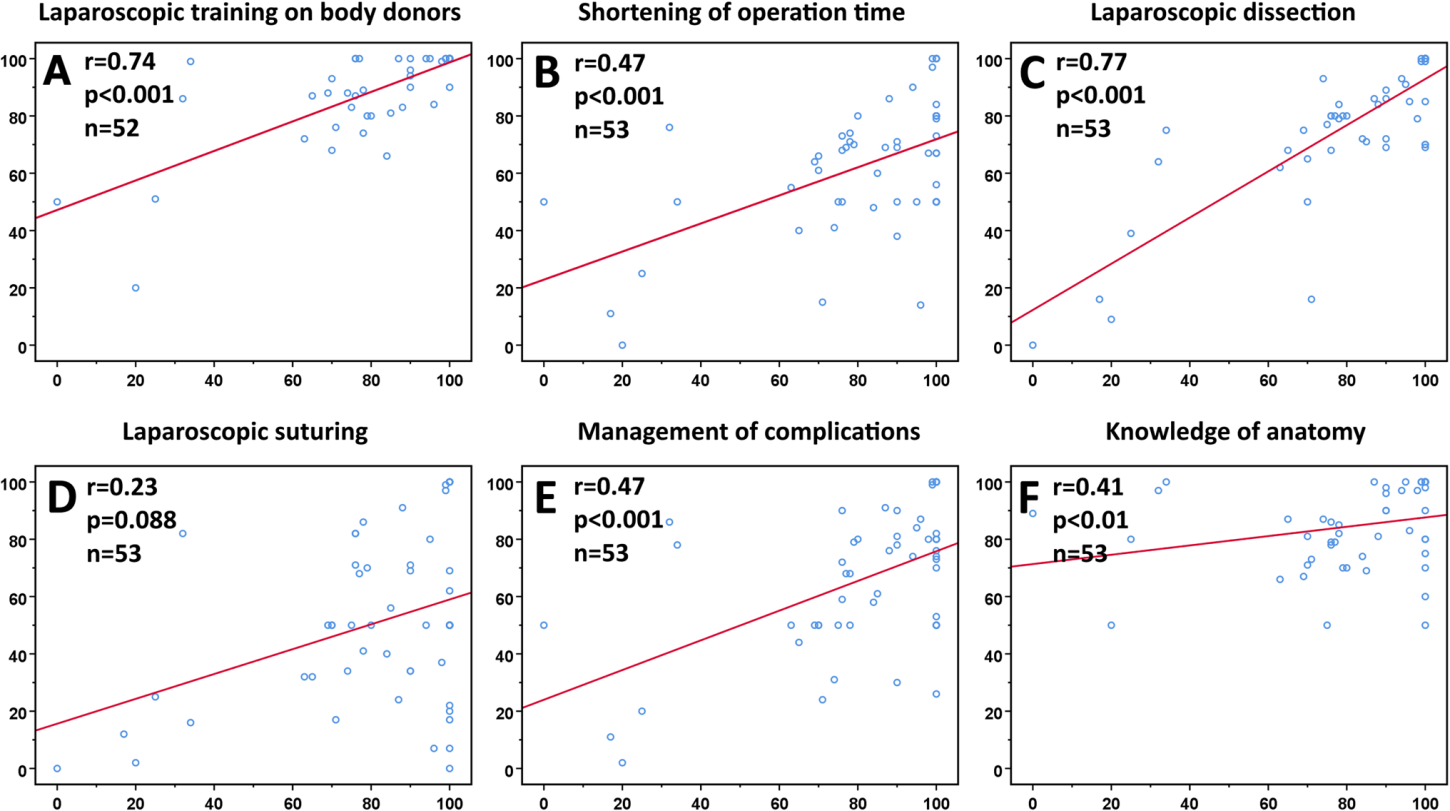
Correlations between different parameters (vertical axis) and the overall benefit of the laparoscopic training course to the daily routine (horizontal axis). Correlation coefficient (*r*) is evaluated as follows: *r* ≤ 0.2 no correlation; 0.2 < *r* ≤ 0.5 weak to moderate correlation; 0.5 < *r* ≤ 0.8 strong correlation; 0.8 < *r* ≤ 1.0 very strong correlation

The perceived benefit of the course to the participants’ daily routine was strongly correlated with the use of body donors (*r* = 0.74, *p* < 0.001, *n* = 52) and the ability of laparoscopic dissection (*r* = 0.77, *p* < 0.001, *n* = 53), moderately correlated with the shortening of operating time (*r* = 0.47, *p* < 0.001, *n* = 53), the improved management of complications (*r* = 0.47, *p* < 0.001, *n* = 53) and the increased knowledge of anatomy (*r* = 0.41, *p* < 0.01, *n* = 53). No correlation was found with the ability of laparoscopic suturing (*r* = 0.23, *p* = 0.088, *n* = 53) (Fig. 4).

## Discussion

While mechanical or virtual trainers, and dead or living animals are valuable training devices, a number of studies have shown the advantages of surgical training on human body donors in terms of tactile conditions, real-life settings and educational benefits [12, 19–24]. The feasibility of laparoscopic gynecological surgery has been tested previously in fresh-frozen, lightly embalmed, or Thiel fixated body donors [25–27]. In particular, the use of either fresh-frozen or Thiel fixated body donors has been successfully implemented into training courses for several years now [28–30].

Ethanol-glycerol-lysoformin fixation is a suitable alternative embalming method of body donors that can be used for laparoscopic surgical procedures [17]. Based on more than three decades of didactic experience [8, 9, 17, 31–38], the Kiel School of Gynaecological Endoscopy developed advanced laparoscopic training courses together with the Institute of Anatomy, University of Kiel, including practical training modules on body donors embalmed by ethanol-glycerol-lysoformin. Over a period of 6 years we were able to show that this embalming technique offers convincing conditions to practice laparoscopic skills and provides a prerequisite for a valuable training concept for advanced laparoscopic surgery. To the best of our knowledge, this survey comprises so far the largest number of participants who have evaluated the benefits and long-term effects of an advanced laparoscopic training course.

Undoubtedly, both Thiel´s embalming method and fresh-frozen body donors provide good tissue properties and a realistic setting [25–27, 29]. Compared to fresh-frozen cadavers, advantages of Thiel’s method include low odor nuisance, the option for multiple reuse and no need for expensive deep-freezing facilities and time-consuming defrosting procedures [28, 29]. However, these advantages were also achieved by using ethanol-glycerol-lysoformin fixation, e.g., low odor, optional reuse, and easy storage [17]. Furthermore,. while the embalming process in Thiel´s method is technically very complex and includes rather expensive chemical compounds [14], ethanol-glycerol-lysoformin fixation is comparatively easy to carry out and cost-efficient [17].

Moreover, the high level of satisfaction of the participants regarding the tissue properties and quality of organs and technical feasibility of realistic laparoscopic procedures further confirms the suitability of this alternative embalming method. Since most participants had extensive professional experience and had attended other laparoscopic training courses, the positive impressions portray a true reflection of the suitability of these body donors for advanced laparoscopic training.

Every practical training method should prove its beneficial effects on the daily clinical routine [9]. Among the different training concepts available, surgical skills courses using body donors yield very high levels of satisfaction of the participants in regard to the educational benefits [25–27, 39]. Chai et al. attributed the pronounced clinical impact of such courses precisely to the strong practical relevance, the realistic reproduction of the clinical setting and, in particular, the quiet and protected environment [28]. In our training setting we could confirm the high degree of satisfaction expressed by the participants not only directly after the course but also 6 months later, giving evidence for the long-term learning effects.

Participants were more likely to report that the course benefited their daily surgical routine if they found useful the use of ethanol-glycerol-lysoformin embalmed body donors and the training of laparoscopic dissection. It became clear that the influence of these training options on the clinical practice was initially underestimated by the participants, who realized the positive effect after they implemented what they learned during the course in their practice. The increased anatomical understanding, a shortening of the operating time and improved complication management also contributed to the practical benefits to their daily routine.

While the above-mentioned parameters correlated well with the overall benefit of the laparoscopic training course to the daily routine, the ability of laparoscopic suturing appeared to be less important in this context. One explanation is that the main focus was on learning anatomy and training specific surgical procedures. Additionally, an advanced laparoscopic training course should be based on the assumption that the participants have previously gained sufficient laparoscopic suturing skills [40], for example by using pelvitrainers which are optimal training devices for suturing techniques [41]. Indeed, most participants in our courses had attended other training courses and had significant clinical experience. However, a standardized documentation of the participants’ previous training experience and skills has not been carried out in detail in previous studies [29, 30, 42] and so we cannot compare our findings with other studies. Thus, it is not possible to state whether the low training effect regarding laparoscopic suturing techniques is due to the participants being already proficient in suturing, the specific set-up of our training course, or to the conditions of the body donors.

Another critical issue is the number of participants per body donor. While our training course involved three trainees per body donor, other concepts reduced the number to two trainees with a remarkably high satisfaction [42]. However, considerable technical and financial efforts are required to offer this generous ratio between trainees and working stations. While costs for the fixation method itself are comparatively low in contrast to Thiel´s method [2, 43], the high technical complexity and associated costs of a laparoscopic training course should be considered. Therefore, implementation of additional training devices (e.g., laparoscopic simulators, bench models) are cost-efficient tools and should complement advanced laparoscopic training courses [44, 45]. In view of the requirements for a high clinical quality standard, this approach seems to be justified in the context of the scarcity of resources and interest of patients´ welfare.

Didactic models have shown that a multimodal training, which enables learning via different educational media, has the greatest possible learning effect [46]. Following this accepted principle, the teaching content of the training course was conveyed by three different modules; (1) visually and theoretically by means of lectures, (2) plastically and three-dimensionally by means of hands-on study of pre-dissected anatomical specimens, (3) haptically and technically by means of laparoscopic procedures on body donors. Similar approaches have been pursued in other body donor courses, and in our training course we particularly emphasized and focused on the in-depth teaching of clinical anatomy [42]. By means of this integrative three-step concept, participants received optimal preparation to maximize the benefit of the final and most challenging module of the training course, the laparoscopic training on body donors. Thus, the time spent at the body donor can be used in the most efficient way for training purposes, thereby ensuring that the body donation enables the highest degree of surgical education.

In summary, this study demonstrates the technical feasibility and high educational potential of the ethanol-glycerol-lysoformin embalming method as a means of performing realistic and sustainable training of laparoscopic skills on human body donors. The feedback from the participants gives evidence that laparoscopic training courses based on body donors are of substantial benefit to the daily surgical routine and should be integrated into structured postgraduate educational curricula to meet both the technical demands of minimal invasive surgery and the ethical concerns regarding patients´ safety.
